# Cryptococcal meningitis in a human immunodeficiency virus-negative patient with rheumatoid arthritis

**Published:** 2016-04-03

**Authors:** Samaneh Haghighi, Maral Seyed Ahadi, Abdorreza Naser Moghadasi

**Affiliations:** 1 Department of Neurology, Sina Hospital, Tehran University of Medical Sciences, Tehran, Iran; 2 Department of Neurology, Sina Hospital AND MS Research Center, Neuroscience Institute, Tehran University of Medical Sciences, Tehran, Iran

**Keywords:** Cryptococcal Meningitis, Cranial Polyneuropathies, Rheumatoid Arthritis

A 68-year-old diabetic female with a 30-year history of rheumatoid arthritis presented to our emergency department with a 2-month history of a headache, blurred vision, and subsequent hearing loss complicated with recent unilateral facial weakness and imbalance.

She has been taking prednisolone 5 mg/day and methotrexate 7.5 mg weekly for almost 20 years. Other medications included oral anti-hyperglycemic agents and calcium supplements.

The patient’s husband, with whom she lives, was diagnosed with tuberculosis (TB) 9 months ago and was treated according to the World Health Organization (WHO) treatment protocol.

Her systemic examination revealed no obvious abnormality. She was afebrile and had neck stiffness, which was performed cautiously regarding the risk of atlantoaxial dislocation, were negative. On neurologic examination, she was alert but not fully cooperative due to profound bilateral hearing loss. Both pupils were reactive to light without evidence of positive relative afferent pupillary defect. Optic discs were blurred in margins bilaterally with reduced visual acuity. Right sided peripheral facial palsy was detected. Deep tendon reflexes and motor forces were reduced globally, and an ataxic gait was notable.

Laboratory investigations revealed a raised total leukocyte count (15700/mm) with 70% neutrophils and normal serum electrolytes, renal function tests, and liver function tests. Human immunodeficiency virus (HIV) antibodies and repeated blood cultures were all negative. The erythrocyte sedimentation rate level was 22; rheumatoid factor and other collagen vascular related tests were negative.^1^ Chest X-ray and lateral cervical X-rays were all normal.

The cerebrospinal fluid (CSF) examination was a cellular with protein and glucose level of 61 and 43 mg/dl, respectively (corresponding blood glucose was 75 mg/dl). Brain magnetic resonance imaging (MRI) with contrast and diffusion-weighted (DW) sequences showed a small deep infarction of the right basal ganglia without obvious evidence of meningeal enhancement or Virchow-Robin space involvement (Figure 1).

During her admission, she became febrile and a second lumbar puncture was considered, which demonstrated raised leukocytes at 40/mm^3^ with a lymphocytic predominance of 80%, low-CSF glucose (27 with a corresponding blood glucose of 110), and raised protein level (62 mg/dl). Regarding her previous TB exposure and recent abnormal CSF analysis, anti-TB drugs were started. After 4 days, the second CSF Indian ink preparation and CSF culture were reported to be positive for Crypyococcus neoformans and we discontinued all anti-TB drugs and initiated long-term treatment with amphotericin B.

Repeated lumbar punctures revealed negative TB CSF culture, TB polymerase chain reaction, Wright, and Coombs-Wright. She continued on amphotericin B liposomal until day 54 when it was changed to fluconazole (200 mg, twice daily). Repeated brain MRIs at 6 weeks showed no new features. The brain MRI remained unchanged, but CSF fungal culture and Indian ink preparation were negative 11 weeks after initiating amphotericin B. CSF analysis revealed mild lymphocytosis with a normal protein and sugar in this stage of the disease. Her condition improved and she was discharged after 14 weeks from admission.

The prevalence of cryptococcal meningitis, most commonly caused by C. neoformans, has increased in the past 20 years due to increased prevalence of HIV infection and use of immunosuppressives.^2^ Its presenting symptoms include fever, headache, fatigue, and a slow progressive mental decline. Frequently there are no symptoms; however, papilledema, meningismus, and cranial nerve palsy may be evident as manifesting signs. Diagnosis is facile in HIV positive patients due to a high fungal load in CSF, which results in a positive Indian ink preparation. However, repeated lumbar punctures and cultures may be indicated to distinguish the causative organism in immunocompetent patients, which was carried out in our patient.^2^

As previously mentioned cranial nerve palsy is a probable presentation of cryptococcal meningitis and may present as sudden or progressive sensory neural hearing, either unilaterally or bilaterally.^3^^-^^5^ However, among the cranial nerves involved, facial nerves seem to be the least reported.^5^ Therefore, the presentation of our patient, including multiple cranial nerves palsies, is completely rare. Previous studies have reported isolated eighth nerve palsy or optic neuropathy; however, the combination of the two with facial palsy is rather uncommon.^5^

**Figure 1:**
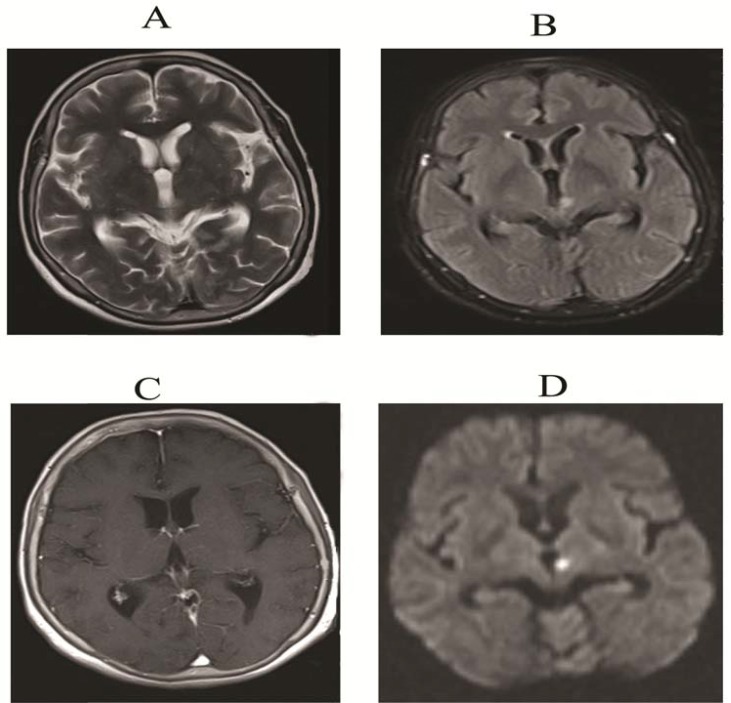
Axial T2 and fluid-attenuated inversion recovery sequences revealed small deep left thalamic hyperintensity (A and B), without any enhancement (C), diffusion weighted sequence also revealed hyperintensity in this area (D)

Krishnamoorthy et al. reported a case of bilateral abducent and facial nerve palsy completely resolved with antifungal treatment as well as degrees of visual acuity and hearing loss resolution were observed.^5^ In our patient, ataxia, facial palsy, headache, and bilateral blurred vision resolved. However, the bilateral hearing loss did not improve.

Cryptococcal meningitis is a rare but fatal complication of immunosuppressive agents. This disease should be considered in all patients receiving cytotoxic presented by cranial nerve palsy.

## Notes:

**How to cite this article:** Haghighi S, Seyed Ahadi M, Naser Moghadasi A. Cryptococcal meningitis in a human immunodeficiency virus-negative patient with rheumatoid arthritis. Iran J Neurol 2016; 15(2): 106-8.
